# Specific inhibition of myostatin activation is beneficial in mouse models of SMA therapy

**DOI:** 10.1093/hmg/ddy382

**Published:** 2018-11-27

**Authors:** Kimberly K Long, Karen M O’Shea, Ramzi J Khairallah, Kelly Howell, Sergey Paushkin, Karen S Chen, Shaun M Cote, Micah T Webster, Joseph P Stains, Erin Treece, Alan Buckler, Adriana Donovan

**Affiliations:** 1 Scholar Rock Inc., 620 Memorial Drive, Cambridge, MA; 2 Myologica, 10811 Dewey Way East, New Market, MD; 3 SMA Foundation, 888 7th Avenue #400, New York, NY; 4 Department of Orthopedics, University of Maryland School of Medicine, Baltimore, MD, USA

## Abstract

Spinal muscular atrophy (SMA) is a neuromuscular disease characterized by loss of α-motor neurons, leading to profound skeletal muscle atrophy. Patients also suffer from decreased bone mineral density and increased fracture risk. The majority of treatments for SMA, approved or in clinic trials, focus on addressing the underlying cause of disease, insufficient production of full-length SMN protein. While restoration of SMN has resulted in improvements in functional measures, significant deficits remain in both mice and SMA patients following treatment. Motor function in SMA patients may be additionally improved by targeting skeletal muscle to reduce atrophy and improve muscle strength. Inhibition of myostatin, a negative regulator of muscle mass, offers a promising approach to increase muscle function in SMA patients. Here we demonstrate that muSRK-015P, a monoclonal antibody which specifically inhibits myostatin activation, effectively increases muscle mass and function in two variants of the pharmacological mouse model of SMA in which pharmacologic restoration of SMN has taken place either 1 or 24 days after birth to reflect early or later therapeutic intervention. Additionally, muSRK-015P treatment improves the cortical and trabecular bone phenotypes in these mice. These data indicate that preventing myostatin activation has therapeutic potential in addressing muscle and bone deficiencies in SMA patients. An optimized variant of SRK-015P, SRK-015, is currently in clinical development for treatment of SMA.

## Introduction

Spinal muscular atrophy (SMA) is a debilitating, frequently fatal neuromuscular disease and the most common genetic cause of infant mortality (1,2). SMA is caused by mutations in Survival of Motor Neuron 1 (*SMN1*) which result in a reduction of the level of full-length SMN protein, sufficient amounts of which are required for survival of the anterior horn cells of the spinal cord. Loss of motor neurons results in profound muscle atrophy, often leading to death due to respiratory insufficiency (3,4). SMA is clinically heterogeneous, with patients categorized into different types of the disease based on the extent and timing of motor milestones achieved. Type 0 is the most severe version of SMA, and is diagnosed prenatally with reduced fetal movement in utero. Patients require ventilator support at birth. Type 1 SMA is typically diagnosed between birth and 6 months, and patients never gain sufficient strength to sit independently. Most Type 1 patients do not survive past 2 years without intervention. Type 2 patients are diagnosed between 6 and 18 months of age. While they are able to sit unassisted, they cannot walk without aid. Patients with SMA Type 3 are diagnosed past the age of 18 months and are able to sit and walk unassisted, although they may become wheelchair-dependent later in life. Type 4 SMA is adult onset, mild in phenotype and very rare (1,5). Although SMA stratification by type is a useful clinical paradigm, the disease phenotype exists more as a continuum than as discrete classifications (5).

The clinical heterogeneity of SMA is due in part to the complicated genetics of the disease. Mutations in the *SMN1* (Survival of Motor Neuron 1) gene result in SMA (6); however, in humans a nearly identical gene, *SMN2*, is located in close proximity to *SMN1 (**7**).* Both genes produce the same protein product, SMN. The primary difference between these genes is a non-coding C–T transition which creates an exonic splice silencer in *SMN2*, resulting in the omission of exon 7 from the mature mRNA transcript. The truncated SMN protein is unstable and quickly degraded. Nevertheless, approximately 10% of the mRNA produced from *SMN2* is correctly spliced and produces full-length SMN protein, although the amount produced is insufficient to fully compensate for loss of *SMN1.* Because the SMN locus is unstable, the copy number of *SMN2* can vary between individuals, with more copies (3–4) generally associated with milder forms of SMA (4,5,8).

Multiple mouse models have been developed to enable preclinical studies of potential SMA therapeutic strategies. Because mice have only a single *SMN* gene, deletion of which is embryonic lethal, various models have been engineered to mimic the human *SMN* locus. The SMNΔ7 mouse, a model of severe SMA, lacks the sole endogenous copy of *SMN*, and expresses human *SMN2*, as well as the human *SMN2Δ7* allele (9). In contrast, the C/C mouse, considered by some to be a milder model of disease, expresses a hybrid gene in which exons 7 and 8 of the endogenous *SMN* gene have been replaced with exons 7 and 8 of human *SMN2*. In addition, this mouse also expresses full-length human *SMN2* (10). The SMNΔ7 mouse, in particular, displays patterns of muscle atrophy and motor neuron loss that more closely mimic the human disease (11).

While a defining clinical feature of SMA is severe skeletal muscle atrophy, not all muscles are equally affected, with proximal muscles displaying generally greater atrophy and denervation than appendicular muscles (11,12). Interestingly, fast-twitch Type II muscle fibers display significantly greater atrophy than slow-twitch Type I fibers (3). The degree of muscle atrophy is directly related to the degree of innervation, with muscles innervated by nerves less affected by SMN loss displaying reduced atrophy (11,13,14). Although muscle atrophy is the predominant symptom of SMA, recent work has demonstrated that patients also show reduced bone mineral density and an increased fracture risk, both in long bones and in vertebrae, a phenotype that is shared with mouse models of the disease (15–17).

The majority of therapeutic approaches in SMA aim to increase full-length SMN protein levels, either in the central nervous system (CNS) or systemically. *SMN1* gene replacement therapy using adeno-associated viral vectors (AAV) has shown benefit in mouse models and in early clinical trials (18–20). Other approaches focus on modulating *SMN2* splicing, such that exon 7 is retained in a greater percentage of transcripts, leading to increased production of full-length SMN protein (21–24). At least two small molecule *SMN2* splice modulators are currently in clinical trials. The most advanced approach is the use of antisense oligonucleotides (ASO; e.g. nusinersen) to block an *SMN2* intronic splice silencer, thus facilitating exon 7 inclusion in both mouse models and clinically (25–30). The demonstrated clinical benefit of this latter approach, including improvements in motor function and compound muscle action potential (CMAP), has led to regulatory approval for its use in individuals with all types of SMA (31). While each of these therapeutic approaches has shown significant pre-clinical and clinical efficacy, significant functional deficits remain in SMA patients following treatment, with the majority of patients significantly impaired compared with their healthy peers (18,26,27,32–37). Recently presented data from the NURTURE trial revealed that patients treated pre-symptomatically with nusinersen are achieving remarkable gains in motor function. Nevertheless, among patients with two copies of *SMN2,* only 4 of 12 could stand unaided and 2 of 8 walk unaided (35). Patients with infantile onset SMA treated with the small-molecule SMN upregulator RG7916 also demonstrated significant gains in function, with 57% of babies achieving CHOP-INTEND (Children's Hospital of Philadelphia Infant Test of Neuromuscular Disorders) scores of ≥40 (out of a maximal score of 64), a milestone essentially never reached in untreated SMA Type 1 patients (34). Gene therapy with AAV-*SMN1* in infantile onset SMA has also had significant effects, with 10 of 12 patients reaching CHOP-INTEND scores of ≥50 and 2 of 12 ≥ 60 (20). While these treatment effects result in significant improvements in patient function, there are nevertheless clear functional deficits remaining, with many patients failing to reach normal motor milestones even with early intervention. Additionally, the majority of patients currently being treated with SMN upregulating therapies have missed the opportunity for early intervention. Treatment of later onset SMA patients with nusinersen post-symptomatically resulted in an increase of 3.9 points in the Hammersmith Functional Motor Scale Extended (HFMSE) score, compared with a 1 point loss in the untreated group. While this is a significant increase in motor function, patients nevertheless continue to exhibit profound motor deficits, with mean HFMSE scores of less than 30 (out of a total score of 66) (33). These post-treatment deficits are reflected in mouse models of SMA, where treated animals display deficits in longevity, body weight, muscle mass and muscle function compared with healthy animals (19,28,29,38). These results indicate that, while therapies that increase full-length SMN expression have the potential for significant effects on SMA disease course and patient quality of life, additional functional gains are needed to further reduce disease burden.

One such therapeutic approach which, either alone or in conjunction with SMN restoration in motor neurons, could improve patient motor function is to prevent the loss of skeletal muscle in individuals with SMA. Treatments that reduce or prevent the muscle atrophy observed in SMA, or promote muscle growth, could offer significant improvements in quality of life. Myostatin (Growth and Differentiation Factor 8; GDF-8) a member of the TGFβ superfamily, is a critical negative regulator of muscle mass and may serve as a potential target for enhancing muscle function in SMA. Genetic loss of myostatin results in significantly increased muscle mass in multiple species, including, in at least one case, humans, and loss of myostatin does not appear to result in any overt detrimental effects (39–42). In preclinical models pharmacologic inhibition of myostatin also increases muscle mass, as well as prevents muscle atrophy in response to limb immobilization, cancer cachexia and corticosteroid treatment (43–46).

Like other TGFβ family members, myostatin is secreted as an inactive precursor, termed proMyostatin, in which presence of the prodomain occludes growth factor access to its receptor. Myostatin activation results from two distinct proteolytic cleavage steps. ProMyostatin is first cleaved by a proprotein convertase, such as furin, that recognizes an RXXR site between the prodomain and the mature growth factor (47,48). Following cleavage, the prodomains remain associated with the growth factor dimer, forming a latent complex (latent myostatin) which remains unable to bind its receptor. Active growth factor is released following a second cleavage by a member of the BMP/tolloid family (such as TLL-2; tolloid-like protein 2) (49). Following these cleavage steps, mature myostatin is released and now able to bind its receptor, thus activating signaling that leads to a reduction in protein synthesis and enhancement of protein degradation (50).

The profound effects of myostatin loss on muscle mass have brought significant attention and effort to this growth factor as a potential therapeutic target for indications in which muscle wasting is a prominent feature, including sarcopenia, cancer cachexia, muscular dystrophies and disuse atrophy (51). The most common approaches to myostatin inhibition have been (1) antibodies that bind to and inhibit the growth factor, (2) antibodies directed against the myostatin receptor, ActRIIB, (3) soluble ligand traps such as ActRIIB-Fc fusion proteins, as well as (4) virally mediated expression of endogenous myostatin inhibitors, such as follistatin (52–56). However, given the high homology between myostatin and other TGFβ superfamily members, particularly GDF11, generation of antibodies specific to myostatin has proven challenging. Additionally, since multiple growth factors signal through ActRIIB, receptor blocking antibodies or ligand traps will inhibit signaling of multiple growth factors (57,58). This lack of specificity has the potential to lead to unwanted side effects, such as the gingival bleeding and telangiectasias observed in patients treated with an ActRIIB-Fc fusion protein, where binding and inhibition of BMP9 activity is thought to be the cause of these adverse events or alterations in follicle stimulating hormones (likely due to activin A inhibition) in patients treated with an antibody directed against ActRIIB (54,59).

Non-selective myostatin inhibitors have previously been tested in the SMNΔ7 and the C/C mouse models. Data from these studies suggest that myostatin may play a role in SMA pathophysiology. Treatment with soluble ActRIIB ligand traps resulted in increased muscle mass in the SMNΔ7 mouse as well as muscle mass and function in the C/C mouse (60,61). Follistatin delivery (by intraperitoneal (IP) injection or transgene) resulted in increased muscle mass in SMNΔ7 mice (61,62). Additionally, AAV-follistatin administered to SMNΔ7 mice treated with a sub-optimal dose of the small molecule *SMN2* splice modulator, SMN-C1, was also effective at increasing muscle mass (38). AAV-mediated overexpression of the myostatin propeptide was also beneficial in the C/C mouse (60). While these agents all inhibit myostatin, none are specific for myostatin inhibition. Follistatin inhibits both activins and GDF11, as does soluble ActRIIB (57,58,63). The myostatin propeptide is also capable of binding to and inhibiting GDF11 (64).

Given the high degree of homology between myostatin and related growth factors and the limited homology between their prodomains, we chose to target the proforms of myostatin to generate antibodies that are highly specific towards myostatin, with no inhibitory activity towards other TGFβ family members. We previously described SRK-015P, a monoclonal antibody which binds to both pro- and latent myostatin and inhibits tolloid-mediated cleavage of latent myostatin (65). SRK-015P does not bind mature myostatin, nor does it bind any form of GDF11 or Activin A or the mature forms of BMP9, BMP10 or TGFβ1. We previously demonstrated that inhibition of myostatin activation is effective at increasing muscle mass and force in healthy mice and preventing muscle atrophy in mice administered dexamethasone (65). Here we demonstrate that muSRK-015P (SRK-015P with a mouse IgG1 framework to reduce the potential for immunogenicity) improves muscle mass and function in two variants of the SMNΔ7 mouse model of SMA which have been pharmacologically adapted to rescue SMN deficiency either at day 1 or day 24, thus representing early or late therapeutic intervention. In addition, we show that inhibition of myostatin activation has beneficial effects on bone, improving both cortical and trabecular phenotypes in these mice. Data presented here suggest that specific blockade of myostatin activation has therapeutic potential for SMA. The optimized progeny of SRK-015P, SRK-015 (65), is currently in clinical development for treatment of SMA.

## Results

### SRK-015P improves muscle mass and function in a model of later SMN restoration therapy

To assess the ability of muSRK-015P to improve muscle mass and function in SMA, we utilized the SMNΔ7 mouse model of SMA, modifying disease severity by treating mice with varying doses of the small molecule *SMN2* splice modulator SMN-C1 (22,38,66). The unmodified SMNΔ7 model is challenging for assessment of therapeutic agents, as the median lifespan of these mice is only 13 days (9). SMNΔ7 mice treated with a low dose (0.1 mg/kg/day; ‘Low’) of SMN-C1 exhibit an intermediate SMA phenotype, but with an extended lifespan, such that approximately 70% of animals survive to day 52. Animals treated with a high dose (3 mg/kg/day; ‘High’) of SMN-C1 exhibit mild functional deficits and near-normal lifespan (38). To emulate therapeutic intervention after disease onset via SMN restoration, mice were treated with low-dose SMN-C1 until postnatal day (PND) 24, at which time the dose is increased to the high dose (0.1 mg/kg–3 mg/kg; ‘Low–High’) (38,67). This variant of the model is valuable in assessing the efficacy of therapeutics that might be used in conjunction with later SMN correction.

**Figure 1:**
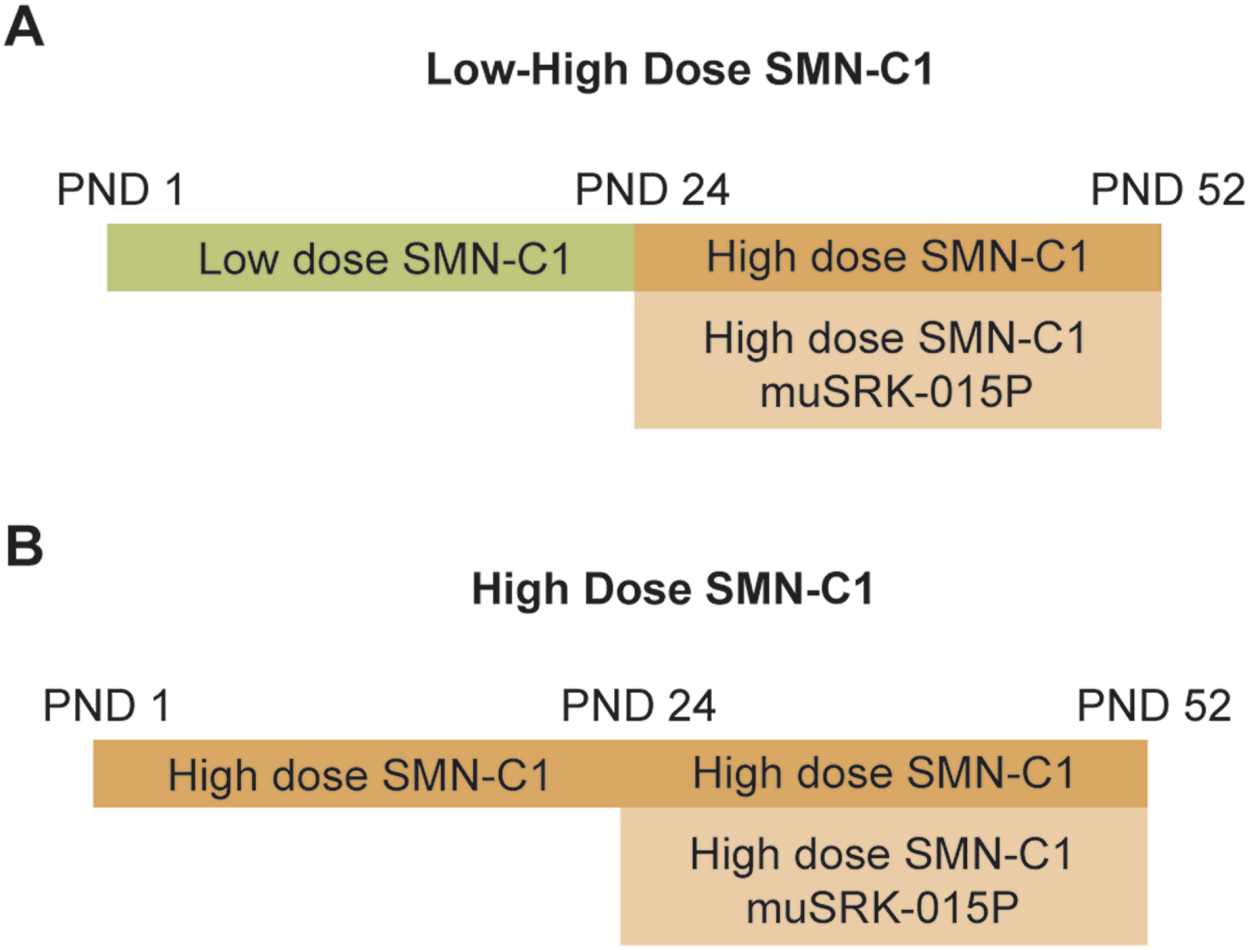
Schematic of SMNΔ7 models. (**A**) For the Low–High-dose SMN-C1 cohorts, mice were administered daily IP injections of 0.1 mg/kg SMN-C1 starting at PND 1. At PND 24 the dose of SMN-C1 was increased to 3 mg/kg/day and weekly injections of vehicle or 20 mg/kg muSRK-015P initiated. At PND 52 functional muscle measures were assessed and muscles collected and weighed. (**B**) For the high-dose SMN-C1 cohorts, mice received 3 mg/kg SMN-C1 by IP injection daily. At PND 24 mice began treatment with vehicle or 20 mg/kg muSRK-015P weekly.

Given that SMN restoration therapy has become the current standard of care for individuals with SMA, we examined whether inhibition of myostatin activation has the potential to improve muscle function in this context. We first assessed the ability of muSRK-015P to improve muscle mass and function in the Low–High model, a translational model for SMN restoration therapy after disease onset. Treatment with 20 mg/kg/week muSRK-015P was initiated at PND 24, concurrent with the switch to high-dose SMN-C1 and continued for 4 weeks (Fig. 1A). This dose of muSRK-015P was chosen as it was previously shown to be fully efficacious in preventing dexamethasone-induced muscle loss (65). Following 4 weeks of treatment, we assessed the *in vivo* force generation of the plantarflexor group (gastrocnemius, plantaris and soleus). Muscles were also collected and weighed, and the plantarflexor group prepared for histology.

**Figure 2:**
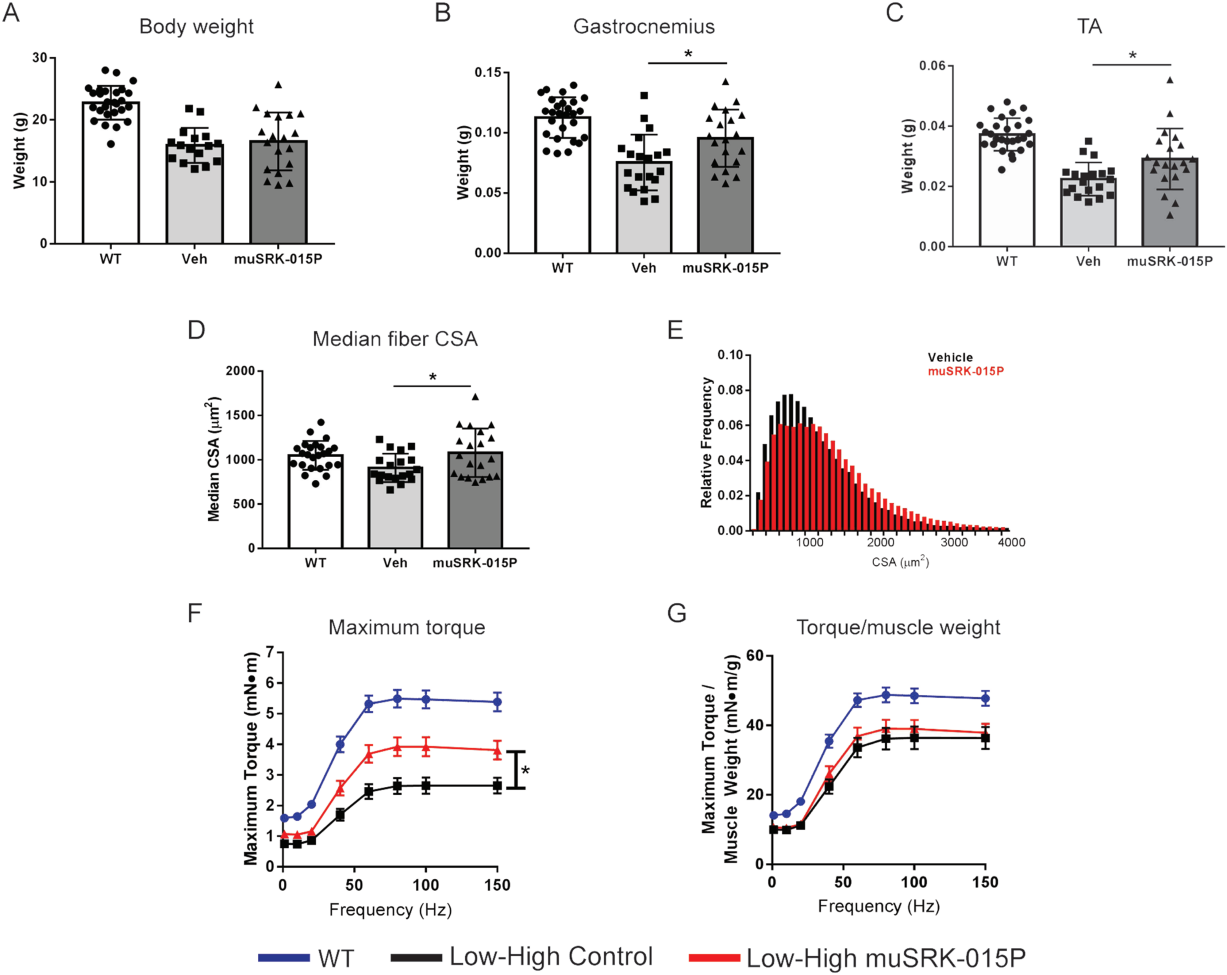
muSRK-015P increases muscle mass and strength in Low–High-dose SMN-C1 treated Δ7 mice. Δ7 mice were treated with low-dose SMN-C1 from PND 1–24. At PND 24 animals began treatment with a high dose of SMN-C1 and were concurrently administered either vehicle or 20 mg/kg muSRK-015P weekly. At PND 52 *in vivo* force measurements of the plantarflexor group were performed and muscles were collected and weighed. Body weights (**A**), gastrocnemius (**B**) and TA (**C**) weights. Both gastrocnemius and TA weights are significantly increased by muSRK-015P treatment. ^*^P = 0.0094 (gastrocnemius) or *P* = 0.014 (TA) compared with vehicle by one-way ANOVA using Tukey’s Multiple Comparison Test. (**D**) Median fiber CSA is increased with muSRK-015P (^*^P = 0.031 by one-way ANOVA). (**E**) CSA distribution of vehicle- and muSRK-015P-treated mice. (**F**) Maximum torque of the plantarflexor group is significantly increased upon muSRK-015P treatment (^*^P = 0.003, repeated measures, two-way ANOVA), while torque normalized to muscle mass is not affected (**G**). N = 27 (WT) or 20 (Vehicle, muSRK-015P). For (D,E) *N* = 19 for Vehicle. Data are mean ± SD (A–D) or mean ± SEM (F,G). **Veh**: Low–High SMN-C1 + Vehicle; **muSRK-015P**: Low–High SMN-C1 + muSRK-015P.

While 4 weeks of treatment with muSRK-015P did not lead to increased body weight (Fig. 2A), treatment did result in a 26.8% increase in the weight of the gastrocnemius and a 29.9% increase in the weight of the tibialis anterior (TA) muscles (Fig. 2B and C). We also observed a 17.8% increase in median total fiber cross-sectional area (CSA), as well as an increased percentage of larger fibers in the antibody treated animals (Fig. 2D and E).While we did not observe an increase in the median CSA of specific fiber types, there was a trend towards increase type IIB fiber CSA (Supplementary Material, Fig. S1). We and others have shown that myostatin inhibition in mice primarily affects type IIB fibers (43, 65). Indeed, analysis of the frequency distribution of IIB fibers shows an increased percentage of larger fibers in the antibody-treated animals (Supplementary Material, Fig. S1). muSRK-015P treatment had no effect on the relative frequencies of different fiber types (Supplementary Material, Fig. S3).

To determine whether the increases in muscle mass observed with muSRK-015P treatment were associated with improved muscle function, the plantarflexor muscle group (gastrocnemius, plantaris and soleus muscles) was evaluated for the ability of muSRK-015P to increase maximal torque *in vivo*. In the SMNΔ7 mouse the gastrocnemius muscle (which makes up the bulk of the plantarflexor group) displays reduced innervation and abnormal nerve terminals (68,69). The plantarflexor group also displays reduced CMAP and motor unit number estimation which can be corrected by treatment with an ASO increasing SMN expression, suggesting that this muscle group is relevant to assess the effects of myostatin inhibition in combination with SMN upregulation (70). As shown in Fig. 2F, vehicle-treated Low–High SMNΔ7 mice display a profound deficit in force. However, animals treated for 4 weeks with muSRK-015P demonstrated a significant 44–51% increase in maximal torque at stimulation frequencies of 40 Hz and greater. When torque is normalized to muscle weight, no differences were observed between vehicle and muSRK-015P treated groups, indicating that the improved function is primarily a result of increased muscle mass (Fig. 2G). Overall, these data indicate that inhibition of myostatin activation significantly improves muscle function in conjunction with SMN upregulation.

### SRK-015P improves muscle mass and function in a model of early SMN restoration

We next assessed the ability of muSRK-015P to improve muscle mass and function in a model of early SMN restoration. For this we turned to the High-dose variant of the SMNΔ7 model, in which animals receive a high dose of SMN-C1 from PND 1 onward. While these mice have significantly improved lifespan, motor neuron preservation, muscle mass and function compared with SMNΔ7 mice treated with low-dose SMN-C1, they nevertheless continue to exhibit deficits in all these parameters when compared with wild-type (WT) animals (38).

**Figure 3:**
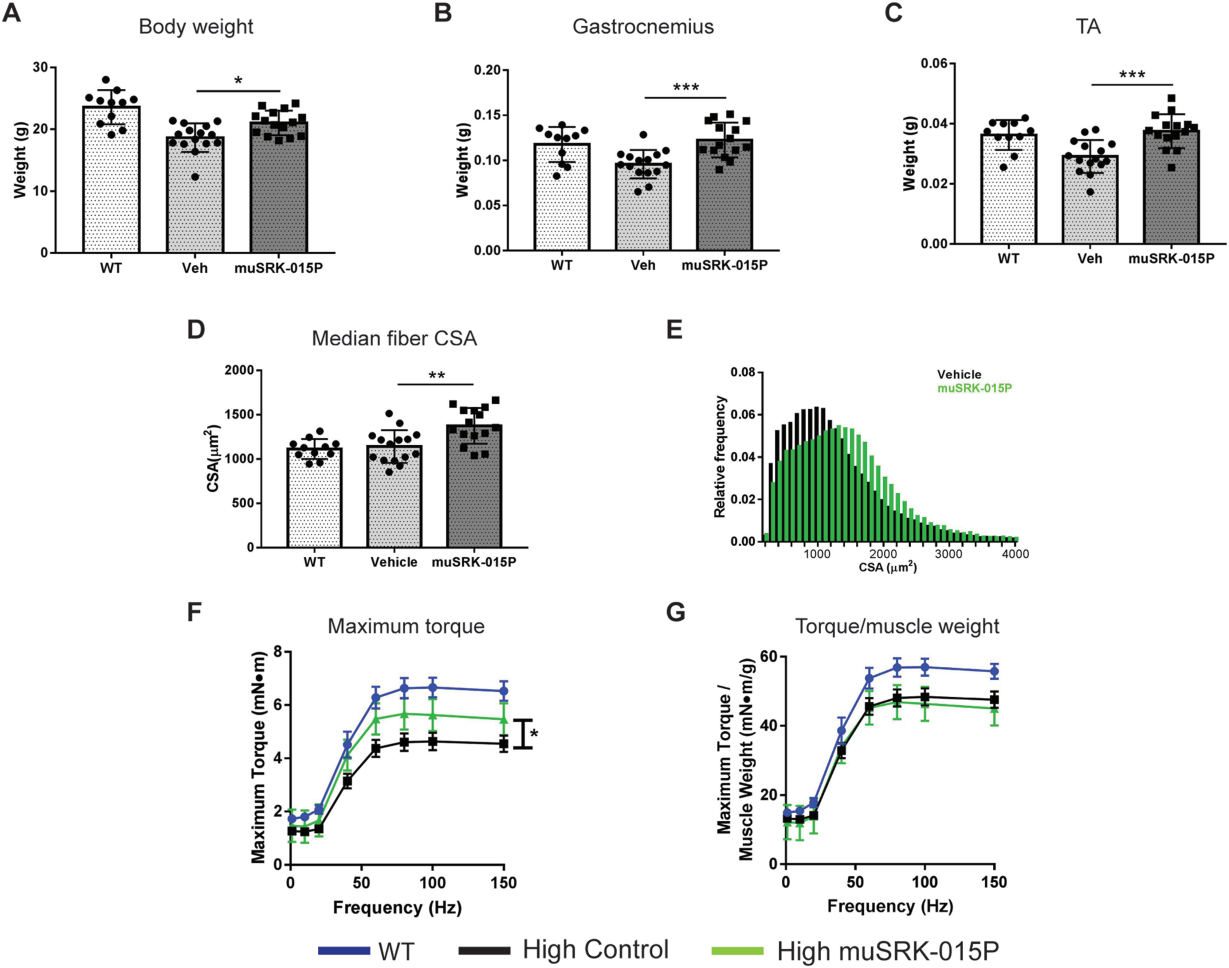
muSRK-015P increases muscle mass and strength in High-dose SMN-C1-treated Δ7 mice. Δ7 mice were treated with high-dose SMN-C1 from PND 1 onwards. At PND 24 treatment with vehicle or 20 mg/kg muSRK-015 weekly was initiated. At PND 52 *in vivo* force measurements of the plantarflexor group were performed and muscles were collected and weighed. Body weights (**A**), gastrocnemius (**B**) and TA (**C**) weights were all significantly increased upon muSRK-015P treatment. ^***^P ≤ 0.0007; ^*^P = 0.02 compared with vehicle by one-way ANOVA using Tukey’s Multiple Comparison Test. (**D**) Median fiber CSA and (**E**) CSA distribution of vehicle and muSRK-015P treated mice. CSA is significantly increased by SRK-015P. ^**^P = 0.0023 by one-way ANOVA using Tukey’s Multiple Comparison Test. (**F**) Maximum torque of the plantarflexor group is significantly increased upon muSRK-015P treatment, while torque normalized to muscle weight is not affected (**G**). ^*^P = 0.025 by two-way ANOVA, repeated measures. *N* = 11 (WT) or 15 (Vehicle and muSRK-015P). Data are mean ± SD (A–D) or mean ± SEM (F,G). **Veh**: High-dose SMN-C1 + Vehicle; **muSRK-015P**: High-dose SMN-C1 + muSRK-015P.

Similar to the Low–High model, High-dose SMNΔ7 mice began treatment with 20 mg/kg/week muSRK-015 at PND 24 (Fig. 1B). Following 4 weeks of treatment muscle weight, function and histology were assessed. Four weeks of muSRK-015P treatment resulted in significant increases in both body weight and muscle weights compared with vehicle-treated animals. Body weight was increased by 14.7% (Fig. 3A), gastrocnemius weight by 28% (Fig. 3B) and TA weight by 31.6% (Fig. 3C). We also observed a significant 16.6% increase in the median fiber CSA, as well as a 25% increase in the median CSA of type IIB fibers (Fig. 3D and Supplementary Material, Fig. S2). The CSA of type I, IIA and IIX fibers was not affected (Supplementary Material, Fig. S2), in line with the known effects of myostatin inhibition on type IIB fibers in mice (43,65). As expected, the CSA distribution of all fibers was shifted rightward, with muSRK-015P-treated mice displaying a greater percentage of larger fibers when compared with vehicle control mice (Fig. 3E). Again, no change in fiber type distribution was noted with muSRK-015P treatment (Supplementary Material, Fig. S3).

Measurement of *in vivo* maximal torque in the plantarflexor group again revealed that muSRK-015P significantly improved muscle function, with antibody-treated animals demonstrating a 20–30% increase in maximal torque at frequencies of 40 Hz and higher (Fig. 3F). When normalized to muscle weight, isometric torque was equivalent between vehicle- and muSRK-015P-treated animals, again indicating that increased muscle function is primarily a result of increased muscle mass (Fig. 3G). These data from both Low–High and High-dose models demonstrate that specific inhibition of myostatin activation effectively increases muscle mass and improves function in models of SMA across a range of disease severities.

### Inhibition of myostatin activation increases cortical and trabecular bone thickness in models of both early and late SMN restoration

SMA has been associated with significant bone loss in mice and humans, with the risk of fractures in SMA patients increasing with disease severity (15–17). Myostatin inhibition or loss of function has been shown to reduce bone loss in other disease models (71–76). To investigate whether inhibition of myostatin activation could improve bone quality in SMN-C1-treated mice, we employed μCT imaging of the tibias from WT, Low–High and High-dose SMNΔ7 mice.

**Figure 4:**
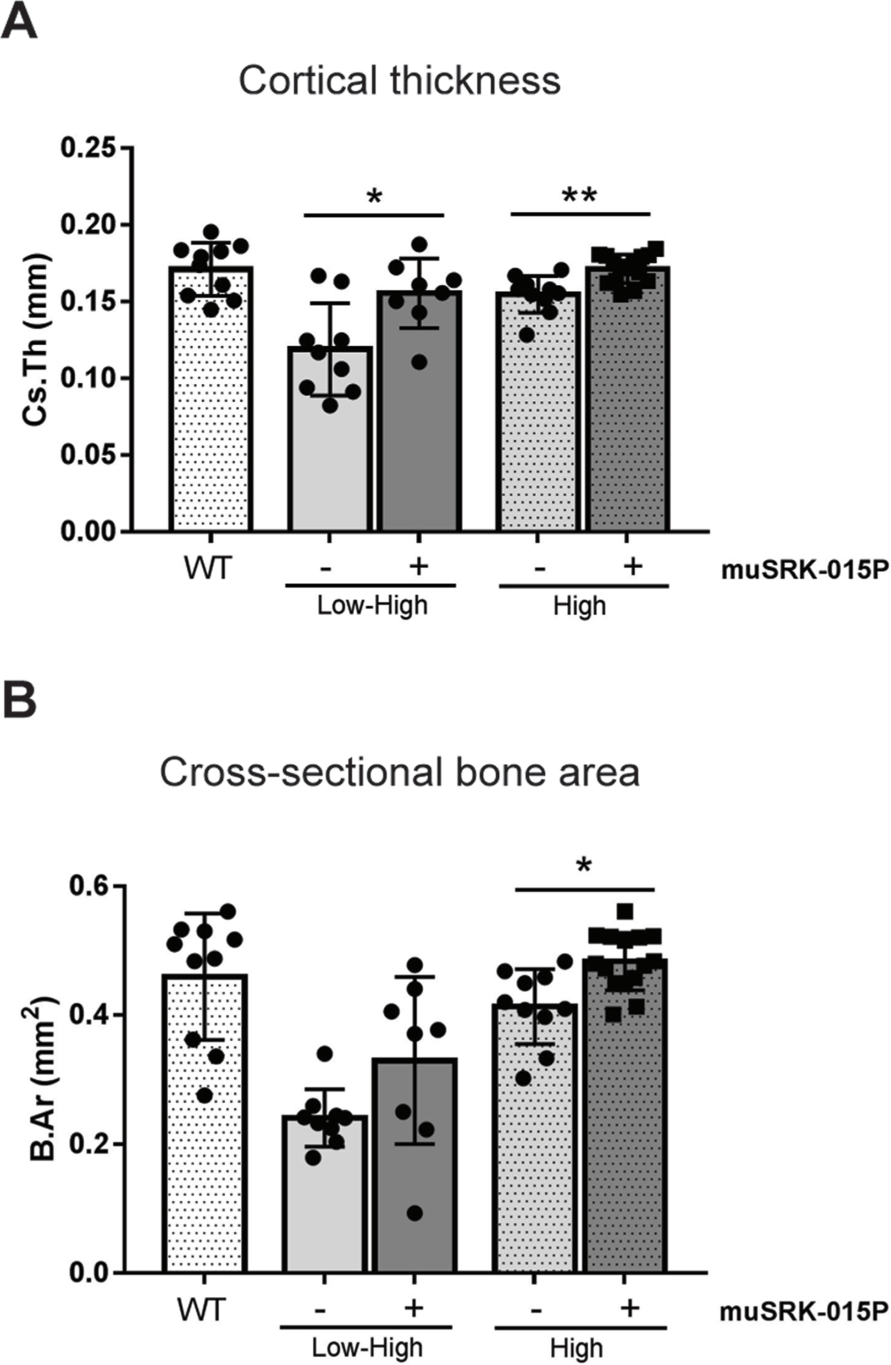
muSRK-015P increases cortical bone thickness in SMN-C1 treated mice. Δ7 mice were treated with Low–High-dose or High-dose SMN-C1. At day 24 treatment with vehicle or 20 mg/kg muSRK-015 weekly was initiated. At day 52 tibias were collected for μCT analysis. (**A**) Cortical thickness. ^*^P = 0.011; ^**^P = 0.009. (**B**) Cross-sectional bone area. ^*^P = 0.04. Significance determined by one-way ANOVA with Tukey’s Multiple Comparisons Test. *N* = 8 (Low–High muSRK-015P), 9 (Low–High Veh), 10 (WT, High Veh) or 15 (High muSRK-015P).

Treatment with muSRK-015P in both SMA models resulted in significant increases in cortical thickness (30.7% Low–High, 10.7% High; Fig. 4A). The deficit in cortical bone parameters at the tibial mid-diaphysis is much more pronounced in the Low–High mice compared with High, as would be expected based on the overall disease severity of these mice. Although Low–High mice treated with muSRK-015P showed trends towards increased cross-sectional bone area, this parameter did not reach significance (Fig. 4B). Inhibition of myostatin activation in High-dose mice did result in a significant 17% increase in cross-sectional bone area (Fig. 4B). These data indicate that muSRK-015P rescued the defects in cortical bone thickness, and the parallel increase in cross-sectional bone area would predict an increase in bone strength.

**Figure 5:**
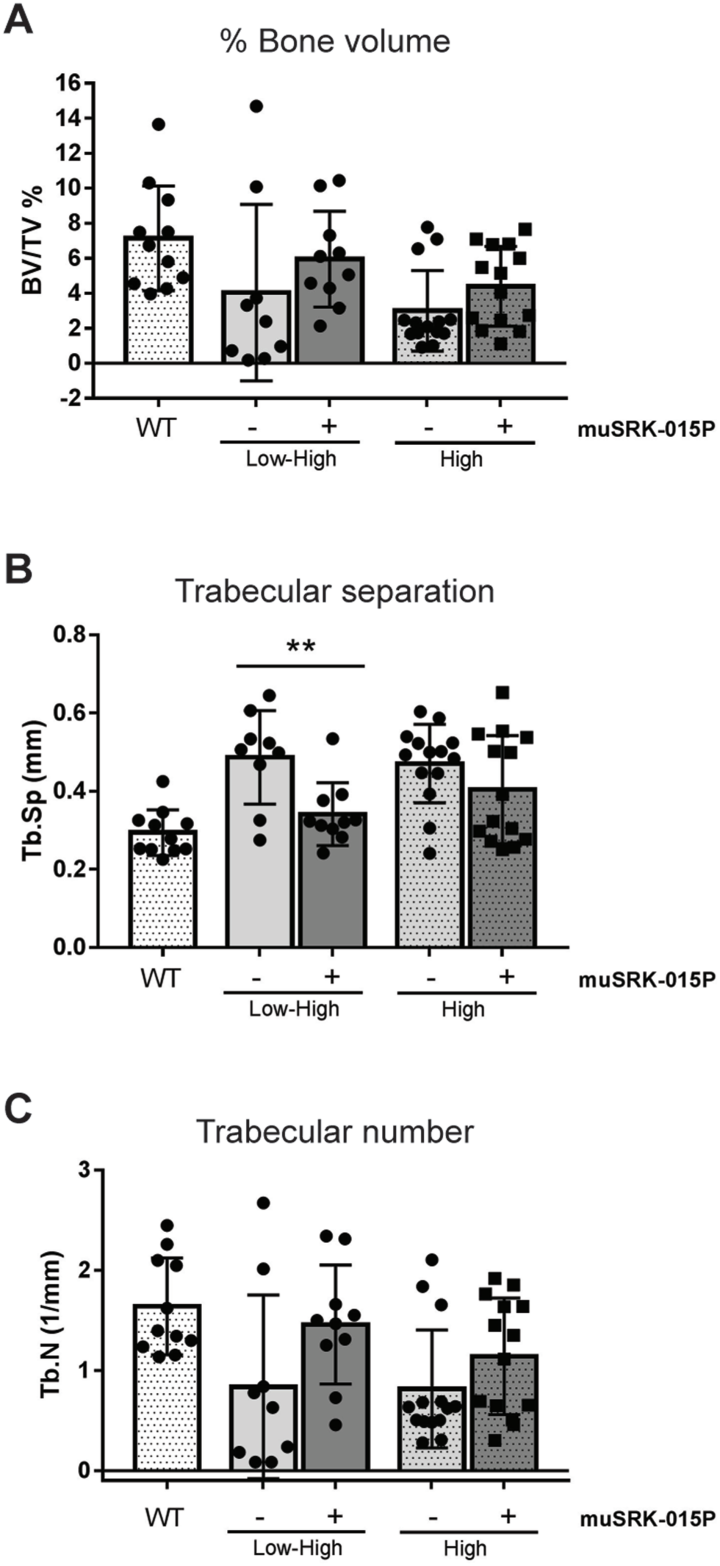
muSRK-015P improves trabecular bone in SMN-C1 treated mice. Δ7 mice were treated with Low–High dose or High-dose SMN-C1. At day 24 treatment with vehicle or 20 mg/kg muSRK-015 weekly was initiated. At day 52 tibias were collected for μCT analysis. (**A**) Percent bone volume. (**B**) Trabecular separation. ^**^P = 0.003. (**C**) Trabecular number. N = 9 (Low–High Veh), 10 (Low–High muSRK-015P), 11 (WT), or 14 (High Veh and muSRK-015P). Significance determined by one-way ANOVA with Tukey’s Multiple Comparisons Test.

We also analyzed tibial trabecular bone in Low–High and High-dose cohorts. While there was no significant increase in percent bone volume, there is a clear trend toward a beneficial effect with muSRK-015P. In vehicle control mice, 7 out of 9 mice in the Low–High group and 11 out of 14 mice in the High-dose group had values below those of all WT mice (Fig. 5A). In contrast, with muSRK-015P treatment, mice given Low–High SMN-C1 show an almost complete reversal of trabecular phenotype, with 8 out of 10 mice having bone volume equivalent to that of WT groups. Similarly, muSRK-015P treatment in High-dose mice also resulted in improvements in bone volume, with 8 out of 14 mice showing values equal to WT (Fig. 5A). In accordance with the bone volume data, muSRK-015P treatment resulted in a significant reduction (30%) in trabecular separation and a trend towards increased trabecular number in Low–High mice (Fig. 5B and C). While neither parameter reached significance in the High-dose animals, there is again a trend toward rescue with muSRK-015P (Fig. 5B and C).

### Target engagement by SRK-015P is equivalent in models of both early and late SMN restoration

Several recent reports have indicated that serum myostatin concentrations are decreased in animals and patients with neuromuscular diseases, raising the possibility that myostatin inhibition is unlikely to be effective in these populations (77,78). To understand the relationship between myostatin levels and disease in our studies, we measured serum latent myostatin levels in all study groups. We and the others have shown that latent myostatin is the predominant myostatin form present in serum (65,79). In addition to assessing latent myostatin in control SMA mice, we also measured latent myostatin from animals dosed with muSRK-015P. This allows determination of target engagement, since binding to muSRK-015P results in accumulation of latent myostatin as the bound target assumes the half-life of the antibody and accumulates in circulation and in target tissues, a phenomenon that has been observed for other antibody, soluble ligand complexes (45,80,81).

**Figure 6:**
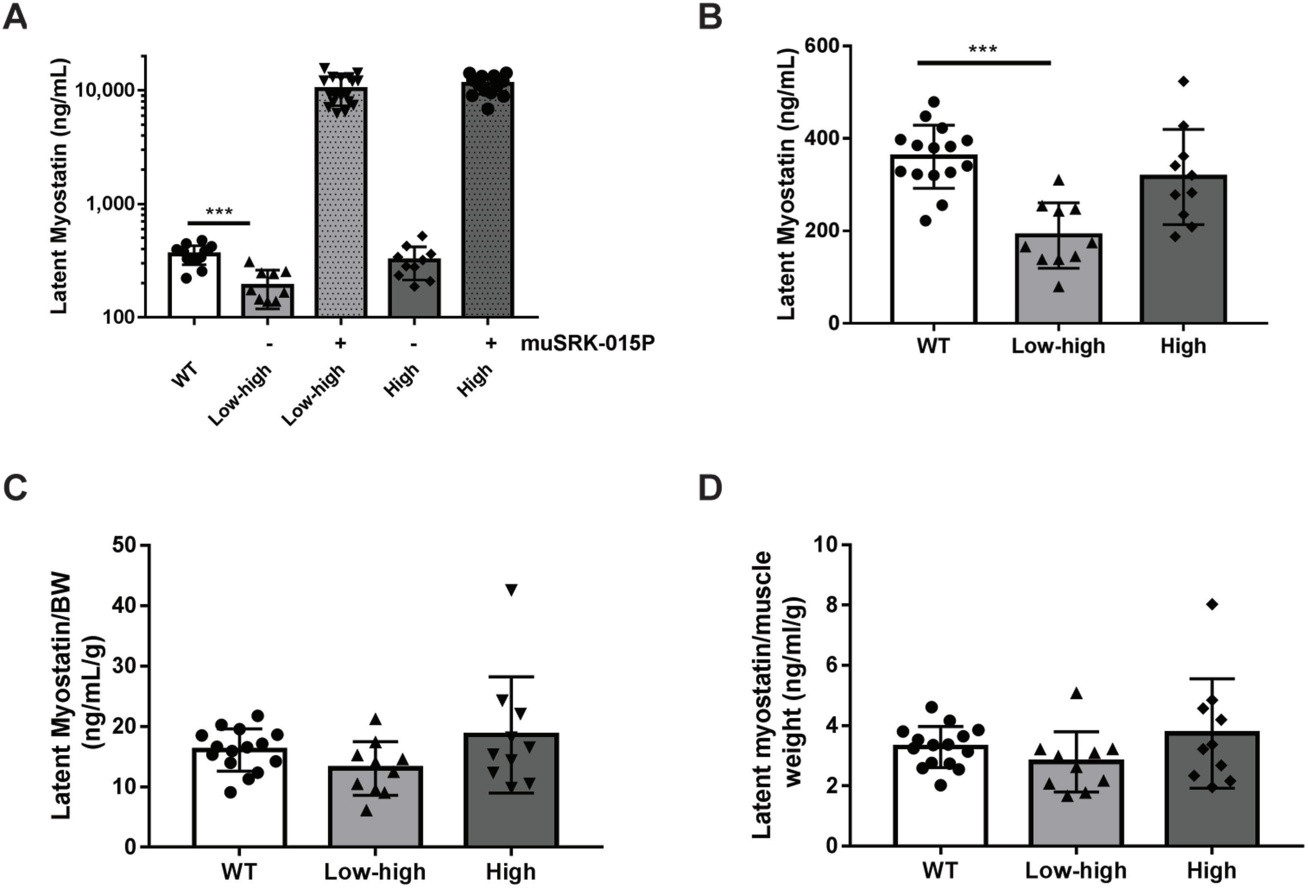
muSRK-015P engages latent myostatin to an equal extent regardless of baseline levels. Serum was collected from WT mice or Δ7 mice treated with the indicated dose of SMN-C1 and vehicle or muSRK-015P. Latent myostatin levels were assessed by ELISA. (**A**) muSRK-015P treatment results in equivalent engagement of latent myostatin and accumulation of target in circulation regardless of baseline levels. Animals treated with Low–High-dose SMN-C1 have significantly reduced serum latent myostatin levels compared with WT mice (A, B). (**B**) shows the control groups only from (A) on a linear scale. However, when normalized to body weight (**C**) or gastrocnemius weight (**D**), baseline latent myostatin levels are equal across all groups. *N* = 15 (WT), 10 (Low–High), 18 (Low–High + muSRK-015P), 10 (High) and 15 (High + muSRK-015P). Data are mean ± SD. ^***^P = 0.0001 by one-way ANOVA with Tukey’s Multiple Comparison Test.

Using a bioanalytical method capable of quantifying latent myostatin over a wide range of concentrations, we observed reduced latent myostatin serum concentrations in control Low–High animals compared with WT (1.9 fold reduction) mice. Latent myostatin levels in High-dose control mice were equivalent to WT (Fig. 6A and B) mice. While decreased latent myostatin levels did correlate with disease severity, we hypothesized that this decrease was due to the reduced muscle mass of these animals. Myostatin is produced in the muscle, and as muscles from patients and animals with neuromuscular diseases are often smaller than healthy muscles, one possibility is that reduced circulating myostatin is simply due to reduced muscle size and not reduced production of myostatin per unit muscle mass. To assess this possibility, we normalized the latent myostatin concentrations to both body weight (Fig. 6C) and gastrocnemius weight (Fig. 6D). In both cases, normalized values were equivalent across all groups, indicating that changes in serum latent myostatin are driven largely by overall muscle mass. This interpretation is supported by the observation that, despite reduced serum latent myostatin levels in the Low–High vehicle control groups, treatment of these animals with muSRK-015P resulted in accumulation of latent myostatin to levels equivalent to those seen in the High-dose-treated animals (10,244 ng/mL versus 11,419 ng/mL), which do not exhibit reduced basal concentrations (Fig. 6A). These data suggest that muscles from the Low–High animals are equally capable of producing myostatin.

## Discussion

Our studies demonstrate for the first time that specific inhibition of myostatin activation effectively increases muscle mass and function in conjunction with both early and later SMN restoration therapy in SMNΔ7 mice. The data presented here support the hypothesis that selective inhibition of myostatin may have therapeutic potential for individuals with SMA, regardless of when treatment with SMN upregulators is initiated. While other molecules that less selectively inhibit myostatin have been shown to have beneficial effects on muscle in murine models of SMA, the contributions of myostatin and non-myostatin inhibition to efficacy by these agents remains unclear (38,60–62). Clinically, non-selective myostatin inhibitors have led to unwanted side effects, such as gingival bleeding and telangiectasias, as well as alterations in hormone levels, believed to be the result of BMP9 and Activin A inhibition, respectively (54,59). These results present a clear rationale for testing the utility of selective myostatin inhibition in conjunction with SMN restoration therapy for the treatment of SMA. Indeed, SRK-015, the optimized clone of SRK-015P, is currently in clinical development for SMA. SMA patients receiving SMN restoration therapy (e.g. nusinersen) will be a major target population.

We assessed the ability of muSRK-015P to improve muscle function in SMNΔ7 mice which received either early or late treatment with the *SMN* splice modulator SMN-C1. Mice were administered an optimal dose of SMN-C1 early (‘High’, PND 1) or later (‘Low–High’, PND 24). In both paradigms, co-administration of muSRK-015P at PND 24 resulted in significant increases in muscle mass and strength, relative to mice with only SMN restoration. The relative increase in strength was greater in the Low–High model compared with the High (44–51% increase versus 20–30% increase). This is likely due to the more pronounced functional deficit in the Low–High mice, thus affording a greater window for potential improvement with antibody treatment. Consistent with our previously reported data, muSRK-015P treatment in both SMN-C1 treatment paradigms also resulted in increased fiber CSA (65).

In addition to profound muscle functional deficits, SMA patients experience loss of bone mineral density, with increased prevalence of fractures in both the femur and the vertebrae (16,17). Similar bone deficits are observed in the Taiwanese (*Smn^−/−^SMN2*) and C/C mouse models (10,15). Here we demonstrate that both the Low–High and High-dose variants of the SMNΔ7 model also display deficits in cortical and trabecular bone and that treatment with muSRK-015P resulted in improvements in both. Cortical bone thickness in particular was markedly improved. Cortical bone was slightly thicker following intervention with significantly more total cortical bone area in High-dose animals treated with muSRK-015P, and a trend toward increased bone area in the Low–High group. While antibody treatment did not result in significant improvements in trabecular bone volume, there was a clear trend towards a treatment effect; the majority of animals in both the Low–High and High-dose vehicle control groups had values below those of WT mice, and with antibody treatment most animals showed a complete rescue of this phenotype. Less selective myostatin inhibitors were shown to be effective in attenuating bone loss in both mouse and non-human primates, but until now it has been unclear whether inhibition of myostatin alone can elicit these protective bone effects (73,75,82). While genetic myostatin deficiency was shown to enhance bone volume, to our knowledge the data presented here are the first examples of a selective myostatin inhibitor having beneficial effects on bone in a therapeutic setting (74,76). It is unclear at this time whether the effects of myostatin inhibition on bone observed in these models are due to direct effects on osteoblasts/osteoclasts, or indirect effects, via increased mechanical load on the bone mediated by larger muscles. The data presented here suggest that, in addition to increasing muscle strength, selective myostatin inhibition has beneficial effects on bone volume and may help to reduce fracture incidence in SMA patients.

In this study we employed a small molecule *SMN2* splice modulator which is administered by IP injection, thereby providing systemic restoration of SMN to SMNΔ7 mice. While several systemic SMN restoration approaches are currently in clinical trials (both small molecules and gene therapy) the only currently approved SMN restoration therapy is an ASO administered via intrathecal injection, affecting SMN expression only in the central nervous system (26). While we have not examined whether administration of muSRK-015P will produce similar muscle and bone benefits in conjunction with a centrally administered ASO, there is reason to support this hypothesis. Studies in the SMNΔ7 mouse model have shown that muscle tolerates much lower SMN levels for proper function than does the CNS (83). Importantly, the functional consequence of SMN restoration in motor neurons (preservation of neuromuscular junctions) is critical for myostatin inhibition to be effective in SMA. Previous work has shown that myostatin inhibition is ineffective at preventing atrophy in the face of complete muscle paralysis, while we have shown that muSRK-015P effectively preserves muscle mass when capacity for minimal physical activity is retained, as is seen in SMA patients (84,85). With motor unit preservation now possible with central SMN restoration therapy, we believe that addition of a myostatin inhibitor is likely to prevent further muscle atrophy and promote muscle growth, resulting in increased functional gains in addition to those already observed with the emerging standard of care. Additionally, there is evidence for positive trophic interactions between muscle and nerve, and we are currently exploring whether improvements in muscle function resulting from inhibition of myostatin activation may have positive impacts in motor neuron connectivity and function (86,87). It is currently unknown whether there exists a degree of motor neuron loss at which the extent of muscle innervation becomes insufficient to facilitate a response to SRK-015. The possibility remains that in muscles more prone to extensive denervation, or in muscles of patients initiating SMN correction later in disease progression, the effects of myostatin inhibition will be less robust than that observed in the mouse models. Of course, these possibilities are likely to be best addressed in clinical studies of selective myostatin inhibition in SMA patients.

Recent data from patients with neuromuscular diseases have indicated that circulating concentrations of myostatin are decreased with disease (77,78). These low myostatin levels may indicate that insufficient target is present for a myostatin directed therapy to be effective. We also observed significantly decreased serum latent myostatin levels in 52-day-old Low–High SMNΔ7 mice. This difference was not observed in High-dose SMNΔ7 mice. However, when we assessed the engagement of latent myostatin by muSRK-015P (by measuring the extent to which latent myostatin accumulates in circulation as it remains bound to antibody), no differences were observed between Low–High and High-dose groups. These data indicate that the muscle of mice with low circulating myostatin is still producing myostatin at a level which allows it to accumulate to the same extent as in mice with no reduction in circulating levels. Additionally, myostatin is produced in the muscle; individuals with reduced muscle mass would be expected to have lower serum myostatin. Indeed, when normalized to body weight or gastrocnemius weight, differences in serum latent myostatin levels are no longer observed. These data suggest that serum myostatin levels are perhaps better viewed as a biomarker for overall muscle content. Since myostatin is produced and acts locally in the muscle, circulating myostatin levels may not be a useful marker for the ability to respond to myostatin inhibition (88). This hypothesis is supported by data from Burch *et al*. (77), in which the authors demonstrate that, while patients with various neuromuscular disorders have reduced serum myostatin, those patients who are ambulatory and presumably have greater muscle mass have significantly higher myostatin levels than those who are not ambulatory.

The recent regulatory approval and advancement of therapies that focus on restoration of SMN protein have provided meaningful benefit to individuals with SMA and are changing the landscape and outlook for people with SMA (18,20,26,27,33,34). Treatment of SMA patients with nusinersen or other SMN upregulators has yielded profound benefits, with some patients reaching all normal motor milestones thus far; however, despite these transformative results, a significant number of patients are not achieving normal milestones, with muscle weakness as a continued unmet medical need (20,27,33–37). It is also clear that additional therapeutic approaches are needed to address the ongoing and significant functional deficits that are not currently met by this emerging standard of care. A primary area of focus will be to address this wide range of severity in functional muscle deficits that remain even with SMN restoration. The work presented here provides evidence that inhibition of myostatin activation in SMA patients may provide therapeutic benefit, particularly to those receiving SMN restoration therapies, regardless of when SMN restoration is initiated. Additionally, inhibition of myostatin activation may be effective as a monotherapy for patients with less severe, but still debilitating, type of disease. We hypothesize that SRK-015 treatment will allow improved muscle growth and function in these patients as neuromuscular junction connectivity is maintained with SMN correctors. Additionally, treatment with SRK-015 may reduce bone loss and fracture incidence by increasing bone volume.

## Materials and Methods

### Antibody production and purification

Transient expression of antibody constructs was carried out in Expi293 cells (ThermoFisher, Waltham, MA, Cat #A14527). Briefly, cells were diluted to 0.6 × 10^6^ cells/mL in Expi293 expression medium (ThermoFisher, Cat# A1435102) and allowed to grow for 3 days (37°C, 8% CO_2_) to reach an appropriate density (2–2.5 × 10^6^ cells/mL) for transient transfection. Heavy-chain and Light-chain DNA were combined at a 1:1 ratio (1 μg total DNA/mL of culture) and 0.20% PEI Max 40000 (Polysciences, Warrington, PA, Cat# 24765) (2.88 μg/mL of culture) were incubated with Expi293 expression medium, separately, for 5 min at room temperature (RT); then combined and incubated for an additional 8 min. These DNA:PEI Max complexes were added to the Expi293 cells cultures. After 5 days, transiently transfected cultures were then harvested via centrifugation at 4000 rpm for 20 min. The supernatants were then sterile filtered and stored at 4°C until ready for purification.

Antibodies were purified using rProtein A Sepharose Fast Flow (GE Healthcare, Chicago, IL) at 10 mg antibody/mL resin. Briefly, rProtein A columns were first equilibrated with phosphate buffered saline (PBS), loaded with clarified and filtered conditioned media, washed with 20 column volumes of PBS, then eluted under acidic conditions using 100 mm Phosphoric Acid, pH 3.0. Fractions containing eluted antibodies were quickly neutralized with 10% volume of 1.6 M HEPES, pH 8.0. NaCl was then added to a final concentration of 100 mM using a 5 M stock solution. Fractions were then analyzed by analytical size exclusion chromatography (SEC). For analytical SEC, 10–20 μg were injected onto a Superdex 200 5 × 150 using 20 mM Phosphate and 200 mM NaCl, pH 6.8 as the mobile phase. Fractions containing eluted antibodies were then pooled and dialyzed into 20 mM Citrate and 150 mM NaCl, pH 5.5. The dialyzed pool was concentrated to 5 mg/mL and filtered. Endotoxin was measured using the Endosafe PTS system (Charles River, Wilmington, MA). All antibodies used in this study were greater than 95% pure with low endotoxin (less than 0.3 EU/mg). Antibodies were then analyzed by analytical SEC and by SDS-PAGE on 4–20% Tris-Glycine gradient gels (Bio-Rad Laboratories, Hercules, CA). Antibody concentrations were determined by UV absorbance using calculated extinction coefficients based on amino acid sequences.

### Mice

Heterozygote SMNΔ7 mice [FVB.Cg-Tg(*SMN*2^*^delta7)4299Ahmb Tg(*SMN2*)89Ahmb] mice were obtained from Jackson Laboratories (Bar Harbor, ME, strain #5025) and bred to generate homozygote SMNΔ7 mice at the University of Maryland Department of Comparative Medicine. Homozygous SMNΔ7 and WT animals were enrolled on study on day 1. Animals were enrolled on a rolling basis with both male and female mice equally enrolled in each group. For Low–High SMN-C1 treated groups, animals were treated with 0.1 mg/kg/day SMN-C1 by IP injection from day 1 until day 23. On day 24, animals began treatment with 3 mg/kg/day SMN-C1 and continued that regimen until testing and sacrifice. For high-dose SMN-C1 cohorts, animals were treated continuously with 3 mg/kg/day SMN-C1 by IP injection from day 1 until study termination. Both cohorts began treatment on day 24 with vehicle or 20 mg/kg/week muSRK-015P (IP) for 4 weeks. Animals were assessed for muscle function and sacrificed on day 52.

### Assessment of *in vivo* muscle force

Muscle performance was measured *in vivo* with a 305**°**C muscle lever system (Aurora Scientific Inc., Aurora, CAN). Animals were anesthetized via inhalation (~5% isoflurane), and placed in a thermostatically controlled table with anesthesia maintained via nose-cone (~2% isoflurane). For assessment of plantarflexor function, the knee was first isolated using a pin through the tibial head and the foot firmly fixed to a footplate on the motor shaft. Contractions were elicited by percutaneous electrical stimulation of the sciatic nerve, and optimal isometric twitch torque determined by increasing the current with a minimum of 30 s between each contraction to avoid fatigue. A series of stimulations were performed at increasing frequency of stimulation (0.2 ms pulse, 500 ms train duration): 1, 10, 20, 40, 60, 80, 100 and 150 Hz, followed by a final stimulation at 1 Hz. Data were analyzed using Aurora Scientific 615 A Dynamic Muscle Analysis Software Suite in high-throughput mode. Each data file was manually inspected to ensure that cursors and fits were assigned properly.

### Bone μCT

Tibias and L6 vertebrae from a subset mice were collected in this study. The decision to assess the effects of muSRK-015 on bone was made partway through the study. Therefore, bones from only 11 WT and 10 Low–High vehicle and muSRK-015P-treated mice were collected. Bones were immersed in 10% formalin for 2 days and then rinsed three times in PBS then transferred to 70% ethanol and kept at RT until analysis. Three-dimensional μCT was performed using a SkyScan 1172 (Bruker, Kontich, Belgium). Tibia and L1–6 vertebrae were scanned with 2 K resolution, 10-micron voxel size, 0.5 mm Al filter at 60 kV and 167 mA. Trabecular bone was delineated manually in a region of interest 0.2 mm–2.0 mm proximal to the distal growth plate. For cortical bone parameters, transverse μCT scans were performed at the diaphysis beginning at 56% of the bone length (measured from the head) extending 0.6 mm distally. The skeletal parameters assessed by μCT followed published nomenclature guidelines (89). Bone morphology and microstructure were assessed at the mid-diaphysis for cortical parameters. Trabecular parameters were assessed at the distal metaphysis. In some cases bones were damaged upon dissection and were unable to be scanned.

### Histology

The plantarflexor muscle group was frozen for histology, sectioned and stained according to standard protocols. For CSA and fiber-type determination, sections were taken from the mid-belly of the muscle and fixed with 4% paraformaldehyde. Sections were then incubated with 0.1% Triton X-100, blocked (SuperBlock blocking buffer, ThermoFisher, cat# 37535B) and incubated with antibodies against MyHC-1, MyHC-IIa or MyHC-IIb (1:20 dilution; BA-D5, SC-71 and BF-F3 antibodies, respectively; Developmental Studies Hybridoma Bank). After washing, sections were incubated with wheat germ agglutinin (WGA) and secondary antibodies conjugated to fluorophores as follows: WGA, Alexa Fluor 633; MyHC-1, Alexa Fluor 488; MyHC-IIa, Alexa Fluor 350; and MyHC-IIb, Alexa Fluor 568 (ThermoFisher Scientific). Sections were digitized using fluorescent microscopy in four channels (Nikon) and the cell boundaries traced using predictive software (Nikon Elements v4.51) via unbiased automated measurements of the entire section, providing CSA of each fiber and total fiber count. Fiber types were determined by ranking the mean intensity of fluorescence of each fiber in each channel. Type IIx fibers were inferred from minimal fluorescence in all channels.

### Latent myostatin ELISA

Since myostatin is endogenously expressed in normal animals, pooled normal mouse serum was treated to deplete any myostatin and create a suitable matrix for preparing standards and controls. A depletion affinity column was prepared through amine coupling of anti-myostatin RK22 antibody directly on functionalized resin (90). A 100 μL volume of the depletion column resin was applied to 25 mL of normal mouse serum and incubated overnight at 4°C with rotation, followed by the addition of two additional 4 h incubations with 100 μL resin. The resin was separated from serum by centrifugation at 1000×g for 5 min. The serum was assayed using the myostatin assay protocol to ensure that the myostatin concentration had been depleted to below the limit of quantitation of the assay. Following this step, standards and controls were prepared in 100% myostatin depleted serum.

Assay plates were blocked with 150 μL/well with a solution of 2.5% bovine serum albumin (BSA) in Tris buffered saline, Tween (TBST). The plates were incubated for 1 h with rotation at RT. The blocking solution was then aspirated from the wells. A 5 μg/mL solution of SR8-C1-biotin was prepared in 2.5% BSA in TBST and added to the assay plate at 50 μL/well. This antibody binds mature and latent myostatin, was generated in house and is based on the sequence of trevogrumab (43). The plate was incubated for 1 h with rotation at RT and then washed 3× with TBST. Serum samples, standards and controls were diluted 1:100 into a solution of 2.5% BSA in 20 mm sodium citrate, 150 mm sodium chloride and 0.01% Tween-20, at pH 4.0. The samples, standards and controls were applied to the plate at 50 μL/well, and the plate was incubated for 2 h with rotation at RT. The assay plates were then washed 3× with TBST. A solution of 0.5 μg/mL of 71B9-ruthenium was prepared in 2.5% BSA in TBST, and 50 μL/well of this solution was added to the plates. 71B9 was identified and produced at Scholar Rock and binds both pro- and latent myostatin. The plates were incubated for 1 h at RT with rotation and then washed 3× with TBST. A solution of 2× Read Buffer T was added to the plates at a volume of 150 μL/well, and the plate was immediately read on a MSD QuickPlex SQ120 plate reader (Mesoscale Diagnostics, Rockville, MD, USA). The concentrations of the standards, controls and samples were generated using the MDS Discovery Workbench (Mesoscale Diagnostics). The standard curve was fitted to a 5-parameter logistic function with 1/Y^2^ weighting.

### Statistics

Muscle torque was compared and assessed for differences using repeated measures 2-way ANOVA with α = 0.05 using SigmaStat v11. Groups were compared using a 1-way ANOVA with Tukey’s Multiple Comparison Test where indicated. *P* < 0.05 was taken for significance. These analyses were performed using GraphPad Prism v.7.

### Study approval

The Institutional Animal Care and Use Committee of the Office of Animal Welfare Assurance at the University of Maryland School of Medicine approved muscle performance studies. Experiments conformed to all Association for Assessment and Accreditation of Laboratory Animal Care guidelines.

## Supplementary Material

Supplementary Data
