# Supplementary material for: Specific inhibition of myostatin activation is beneficial in mouse models of SMA therapy
Source: Hum Mol Genet. 2018 Nov 27;28(7):1076–89. doi: 10.1093/hmg/ddy382 (PMC6423420; doi:10.1093/hmg/ddy382)
Supplement: Supplementary Data [file ddy382_suppl_data.zip › Long, et al., HMG supplement.docx]

**Figure S1**: **muSRK-015P treatment of low-high dose SMN-C1 mice does not affect fiber number or CSA of specific fiber types**. Histological analysis of the plantarflexor group from WT and vehicle or muSRK-015P treated Δ7 mice. (A) Fiber number. (B) Type IIB median CSA. (C) Type IIA median CSA. (D) Type IID median CSA. (E) Type I median CSA. (F) Frequency distribution of Type IIB fibers. N=27 (WT), 19 (Vehicle) or 20 (muSRK-015P).

**Figure S2:** **muSRK-015P increases Type IIB CSA in high dose SMN-C1 treated Δ7 mice**. Histological analysis of the plantarflexor group from WT and vehicle or muSRK-015P treated Δ7 mice. (A) Fiber number. (B) Type IIB median CSA. *P=0.0016. (C) Type IIA median CSA. (D) Type IID median CSA. *P<0.025. (E) Type I median CSA. (F) Frequency distribution of Type IIB fibers. N=11 (WT) or 15 (vehicle and muSRK-015P). Significance determined by One-Way ANOVA.

**Figure S3: muSRK-015P does not alter fiber type composition.** Fiber type composition was determined of the plantarflexor group by immunofluoresence. Data are presented as percent of total fiber number.
