# Supplementary figures and images for: Specific inhibition of myostatin activation is beneficial in mouse models of SMA therapy
Source: Hum Mol Genet. 2018 Nov 27;28(7):1076–89. doi: 10.1093/hmg/ddy382 (PMC6423420; doi:10.1093/hmg/ddy382)

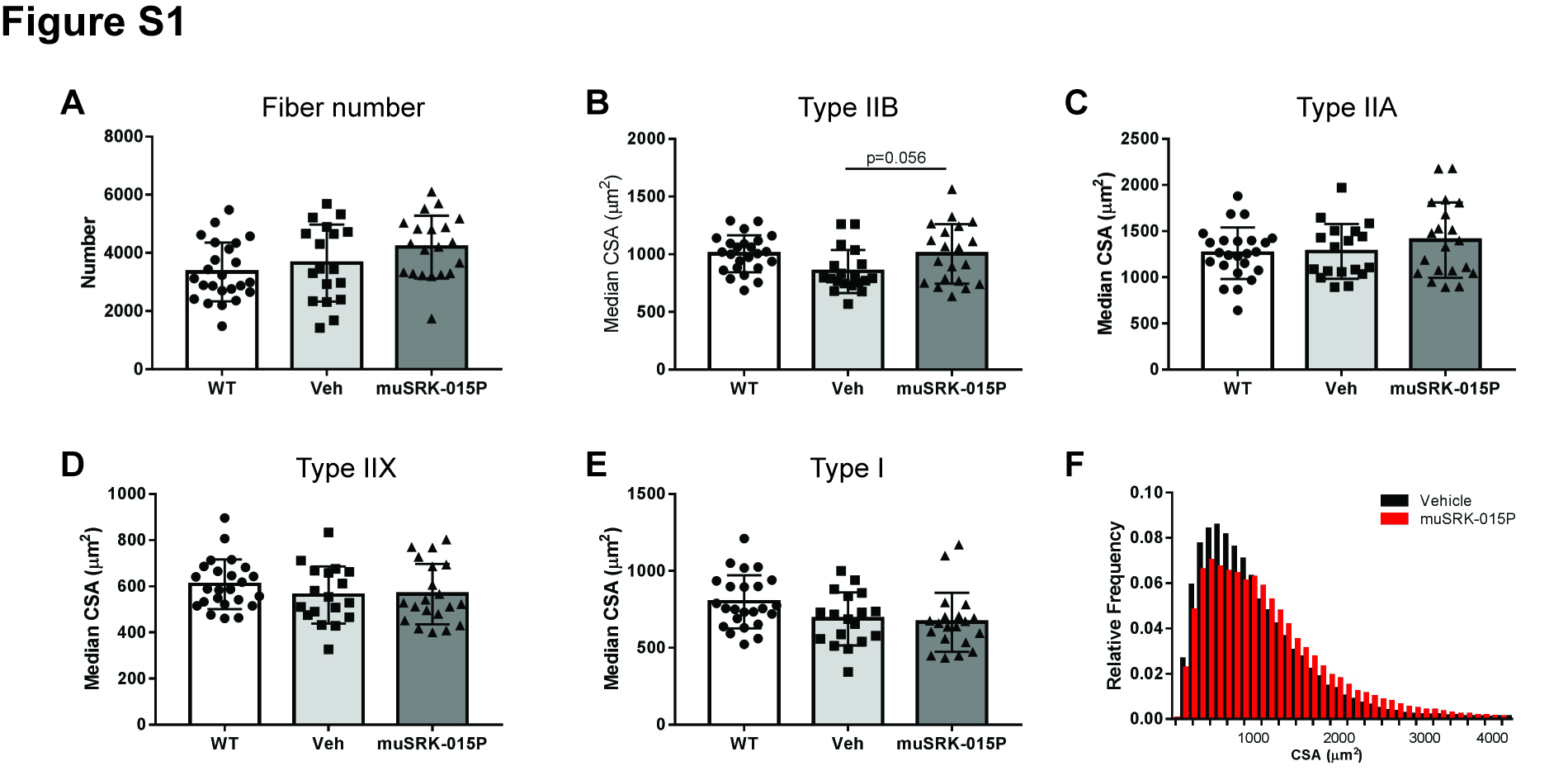

Supplement: Supplementary Data [file ddy382_suppl_data.zip › Figure S1.tif]

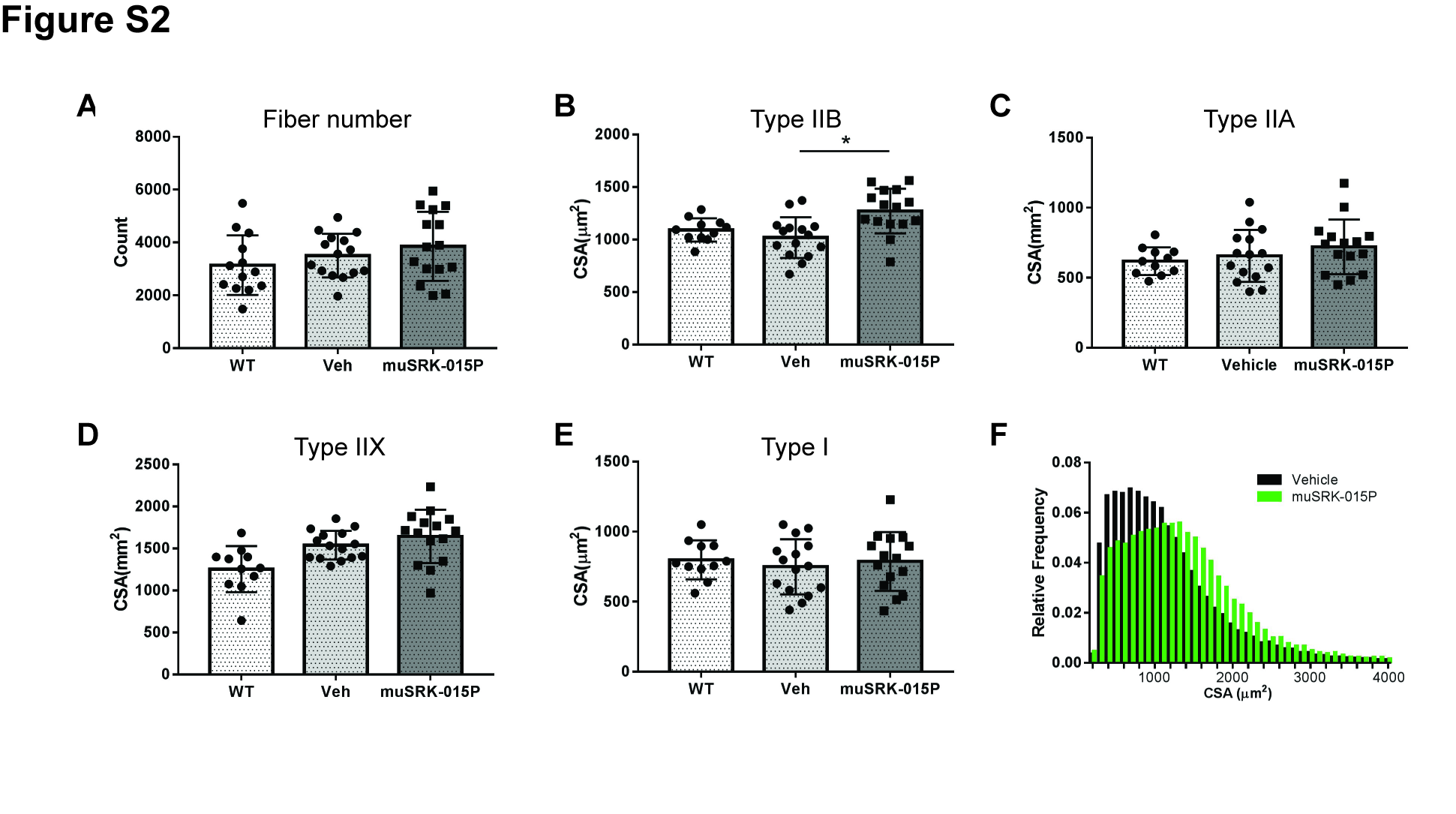

Supplement: Supplementary Data [file ddy382_suppl_data.zip › Figure S2.tif]

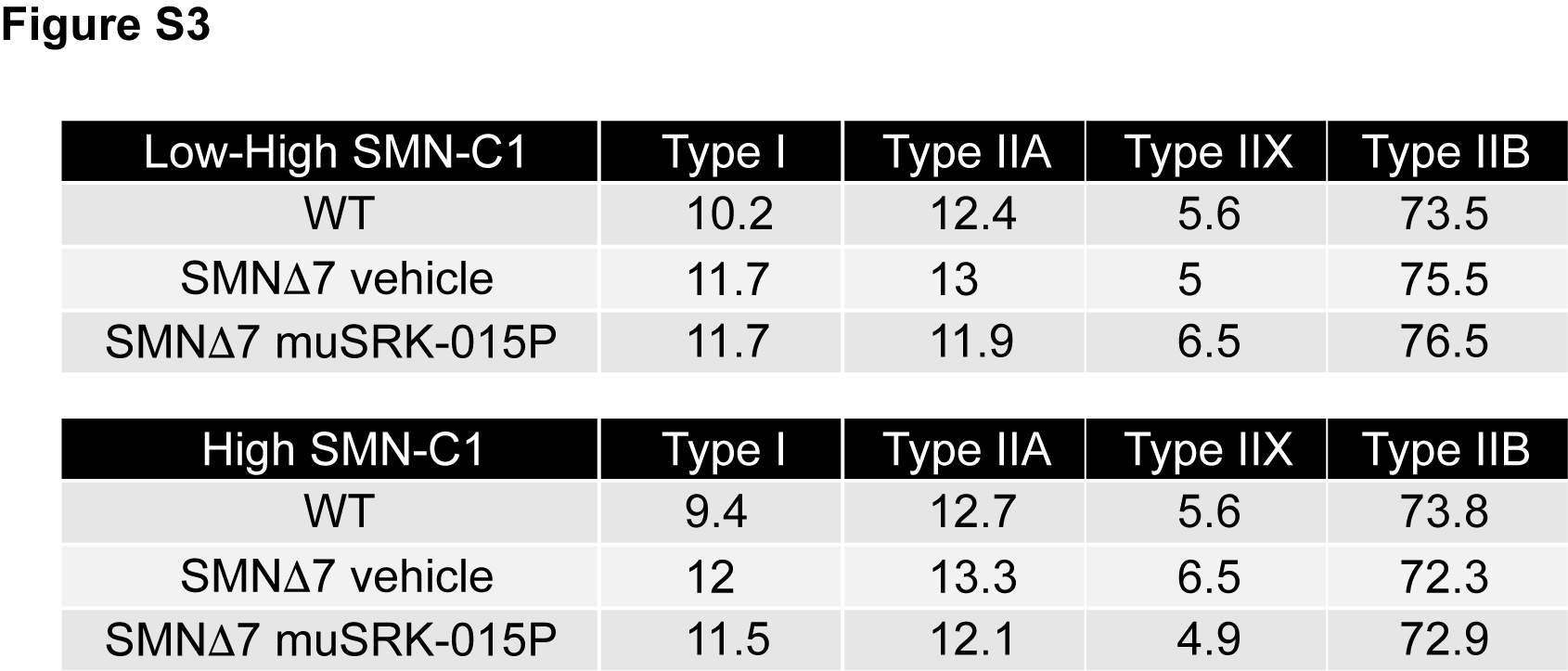

Supplement: Supplementary Data [file ddy382_suppl_data.zip › Figure S3.tif]
